# Strategies of tenogenic differentiation of equine stem cells for tendon repair: current status and challenges

**DOI:** 10.1186/s13287-019-1291-0

**Published:** 2019-06-18

**Authors:** Asiyeh Shojaee, Abbas Parham

**Affiliations:** 1 Division of Physiology, Department of Basic Sciences, Faculty of Veterinary Medicine, Ferdowsi University of Mashhad, Mashhad, Iran; 2 Stem Cell Biology and Regenerative Medicine Research Group, Institute of Biotechnology, Ferdowsi University of Mashhad, Mashhad, Iran

**Keywords:** Horse, Tendinopathy, Cell therapy, Stem cell, Cell differentiation, Tissue engineering

## Abstract

Tendon injuries, as one of the most common orthopedic disorders, are the major cause of early retirement or wastage among sport horses which mainly affect the superficial digital flexor tendon (SDFT). Tendon repair is a slow process, and tendon tissue is often replaced by scar tissue. The current treatment options are often followed by an incomplete recovery that increases the susceptibility to re-injury. Recently, cell therapy has been used in veterinary medicine to treat tendon injuries, although the risk of ectopic bone formation after cell injection is possible in some cases. In vitro tenogenic induction may overcome the mentioned risk in clinical application. Moreover, a better understanding of treatment strategies for musculoskeletal injuries in horse may have future applications for human and vice versa. This comprehensive review outlines the current strategies of stem cell therapy in equine tendon injury and in vitro tenogenic induction of equine stem cell.

## Background

Tendons are dense connective tissues that connect the muscles to the bones and transfer forces generated by the muscles to the bones for locomotion. The extracellular matrix (ECM) of the tendon, which is mainly composed of type I collagen fibrils, is turned over by tenocytes which are responsible for the synthesis of collagen and extracellular matrix components [1]. Tenocytes, being terminally differentiated, are elongated cells with extended nuclei within the fascicles. In addition to tenocytes, tendons contain stem/progenitor cells (TSPCs) as roundly shaped with ovidal nucleus between the fascicles. In general, the location of different types of TSPCs is poorly understood and defined but they are most likely limited to endotenon and adjacent to the vasculature [2]. A number of critical transcription factors in tendon development and differentiation have been identified. Scleraxis (SCX) is required for the generation of tendon progenitors whereas Mohawk (MKX) and early growth response 1 and 2 (Egr1/2) are involved in tendon differentiation and the regulation of genes encoding tendon-specific ECM proteins such as *COL1*, *COL3*, and *TNMD* [3, 4].

It has been reported that up to 46% of musculoskeletal injuries are tendon injuries including tendinopathy [5]. Most of the tendinopathy cases have been caused by a combination of intrinsic and extrinsic factors, including age, gender, disease, occupation, and physical training. Tendinopathy consists of a series of reactions caused by physical overuse. If physical overuse persists, eventually, a defective healing response to accumulated micro-injuries led to degenerative tendinopathy. Persistent hypoxia is one of the major drivers of tendinopathy following the upregulation of expression of vascular endothelial growth factor (VEGF) which induces the expression of matrix metalloproteinases (MMPs) resulting in degradation of the tendon matrix [6]. Recently, it has been increasingly accepted that inflammation and degeneration may not be considered to be two separate processes in tendinopathy. Tendinopathy can be classified as either acute, due to excessive overload, or chronic, due to degenerative condition that is persistent over time [7]. A tendinopathy therefore can include tendon injuries such as paratenonitis, tendonitis, and tendinosis [8].

Injury of superficial digital flexor tendon (SDFT) is one of the most frequent causes of lameness and wastage in racehorses [9]. The process of tendon healing is slow; this poor healing ability happens due to its hypo-vascularity in tandem with hypo-cellularity. The scar formation and ectopic mineralization after tendon injury can induce rupture in the tendon of predisposed horse and happen through increased expression of collagen type III (COL3) that has smaller fibers and fewer crosslink compared to collagen type I (COL1) leading to inferior mechanical properties [10, 11]. The current treatment options result in pain relief or replacement of the injured tissue that remained as a clinical challenge to achieve a functional tissue. In recent years, stem cell therapy has received increasing attention as an alternative therapeutic option. The identification and characterization of appropriate sources of cells are required to achieve more effective repair or regeneration of injured tendons*.*

The objective of the current review is presenting a summary of recent studies in order to inform the reader firstly about various aspects of stem cell therapy for tendon injury in horse and secondly about the current strategies for defining the optimal conditions for in vitro equine tenogenic differentiation.

## Use of undifferentiated stem cells for repair of tendon injury

### Bone marrow-derived MSCs

In 2003 for the first time, Smith et al. implanted 6.4 × 10^5^ of autologous bone marrow-derived mesenchymal stem cells (BM-MSCs) into SDFT of horse that had suffered a strain-induced injury. There was no observable swelling of the limb and no lameness at the walk; also, the ultrasonographic images revealed the lesion filled with granulation fibrous tissue and no adverse effects in healing tendon was observed; the case study opened the door to further researches. Although most of the clinical investigations has shown promising results of BM-MSC injection into SDFT defects, there are possible disadvantages including painful procedures of BM harvesting, long periods of cell expansion, increasing donor age, passage number that reduces differentiation potential, and possible bone formation following BM-MSC injection [12, 13]. Moreover, the injection of large volumes of BM not only contains a small number of MSCs but also might disrupt the intact tendon tissue [12].

### Adipose tissue-derived mesenchymal stem cells

Adipose tissue-derived MSCs (ASCs) are the most abundant and accessible source of MSCs. In addition, yielding higher numbers of MSCs derived from equivalent amounts of fat versus bone marrow provides another advantage in using ASCs. ASCs have also attracted great attention as the best candidate for cell therapy due to their ability to produce and secret ECM component and cytokines [14]. For the first time in an animal trial study, the positive influence of ASCs on tendon repair in horse is reported by Nixon et al. [15] as described in Table 1. Moreover, ASCs showed the greatest expression of the component of tendon ECM in comparison with MSCs from other sources and may be a promising cell source for the treatment of equine tendinopathy [33].

**Table 1:** Summary of in vivo studies of cell therapy for tendon injury in horse

Cell source and injected cell number	Supplement	Follow-up	Evaluation	Observation	Pros/cons	Reference
BM-MSCs 5 × 10^6^ in 1 ml	–	3 years	Comparison with 2 large study with the same follow-up but treated in other ways for 141 horses with natural model injury (overstrain)	No side effects; reduction of the re-injury rate	Long-term efficacy of MSCs/not include the contralateral limb	[16]
BM-MSC 10 × 10^6^ in 2 ml	BM supernatant	3 months	Comparison of the effect of supernatant alone or with cell on collagen fibril size and tensile strength (surgical model)	No difference in collagen fibril diameter and strength between control injury and treated injury	The surgical model for tendon injury induces standardized traumatic fiber damage/the surgical model does not represent certain aspects of natural injury	[17]
ADNC	–	6 weeks	Short-term efficacy of ADNC fractions for 8 horses with collagenase-induced tendinitis	Improved tendon organization and *COMP* expression in treated tendons	Cons: long-term studies are needed	[15]
ASCs 10 × 10^6^ in 0.5 ml	–	120 days	Effect of cell therapy for 8 horses with collagenase-induced tendonitis	No adverse effects; minimal cellularity; parallel arranged extracellular matrix similar to normal tendon; greater collagen deposits compared with the control group	Cons: long-term studies are needed, and biomechanical and genetic expression analyses are needed	[18]
ASCs 10 × 10^6^ in 1 ml	PC	16 weeks	Effect of AD-MSCs combined with PC for therapy of 8 horses with collagenase-induced tendonitis	Greater organization; decreased inflammation; increased blood flow; no difference in the expression of the *SCX*, *TNMD*., *COL* 1 and 3, and *TNC* between the control and treatment groups	Double centrifugation for the collection of the PC/non-activated PC	[19]
ASCs 1 × 10^6^ in 5–10 ml	PRP	9 months	Effect of single injection of cells in 9 athletic horses with spontaneous and acute lameness of SDFT	Decrease in the size of the lesion after 60 days; full alignment of tendon fibers after 120 days; seven horses resumed their normal competitive activity after 7 or 9 months; two horses had relapsed	Pros: rehabilitation program after cell therapy	[20]
Allogeneic ASCs 2 × 10^6^ in 1 ml	PRP	24 weeks	Safety and efficacy of a therapy of 19 horses with acute (less than 10 days old) or sub-acute (less than 20 days old) overstrain SDFT injury	No immune response existed; 89.5% of the horses returned to their previous competing level	Rehabilitation program/no control group was included; higher number of animals; histological, biochemical, and biomechanical data is required	[21]
ASCs 10 × 10^6^ in 2 ml (1.5 ml injected)	–	Up to 9 weeks	Potential low-field MRI to monitor the fate of cells labeled with SPIO nanoparticles (surgical model tendinopathy)	High numbers of cells were present in lesion site	Small number of horses were included; controlled clinical trials are needed; monitoring for a longer time is needed	[22]
Labeled ASCs 10 × 10^6^ in 1 ml	Serum	24 weeks	Long-term cell tracking of MSC after local application into tendon lesions and its effect on tendon healing (surgical procedure with collagenase application)	Part of cells appeared to remain viable and integrated within the injured tissue; no difference between MSC-treated tendons and the serum-injected controls at 24 weeks	MRI is an advantageous for long-term tracking/MRI is not suitable for systemic distribution of labeled cells; SPIO-induced hypointense artifacts. Exact percentage of cells surviving is needed	[23, 24]
Allogeneic UCB-MSCs 2–10 × 10^6^ in ml		6 months	Therapeutic effect of repeated injection UCB-MSCs on tendon and ligament of 52 horses; natural core lesion/anechogenic diffuse lesion	77% (40 horses) regained their higher level of performance	Cons: lack of a sufficient control group	[25]
oAECs 7 × 10^6^ in 0.5 ml	–	18 months, 180 days	Efficacy of healing process in fifteen horses with acute tendon lesions; efficacy of regeneration in acute and chronic lesion	Any adverse reaction to oAEC xenotransplantation and 12 horses resumed competition and their previous activity after 18 months; outcome was similar in both acute and chronic lesions after 180 days	Long-term follow-up/optimal number of injected cells and higher number of chronic cases is required	[26, 27]
BM-MSC and ESC 1 × 10^6^ in 0.5 ml	–	3 months	Monitor survival of injected cells into lesion (surgical model)	BM-MSC survival was less than 5% after 10 days; ESC numbers were at a constant level for 90 days in the absence of tumorigenesis	Two different labels which are used to detect the 2 cell types; not able to compare their detection efficiencies due to different sensitivities	[28]
MSC and IGF -I gene-enhanced MSC 10 × 10^6^ in 1 ml	–	8 weeks	Evaluated for biochemical composition and mechanical test; collagenase-induced lesions	No different effect between both of cells	Cons: optimal dose of MSCs, extended IGF-I expression and less viral vectors for IGF-I delivery should be investigated	[29]
Tenogenic induction allogeneic Pb-MSCs 2–3 × 10^6^ in 1 ml	PRP	2 years	Safety and clinical efficacy for 6 week; long-term efficacy of a combination of PRP and MSCs to treat natural tendon injury	No adverse effect; no calcification; low re-injury rate after 2 years (18% vs 44%)	Cons: no control groups were included; veterinary practitioners for scoring were not blinded	[30, 31]
TSPCs 5 × 10^6^ in 0.15 ml at 2 sites (1 × 10^7^ cells in total)	–	16 weeks	Evaluate the efficacy of autogenous TSPC injections in a collagenase-induced model injury	Improved the tensile strength and collagen fiber alignment	Cons: long-term effect of TDPCs on the biomechanical properties will be determined	[32]

*Abbreviation*s: *BM-MSCs* bone marrow-derived mesenchymal stem cells, *ASCs* adipose tissue-derived MSCs, *ESCs* embryonic stem-like cells, *ADNC* adipose-derived nucleated cell, TSPCs tendon-derived progenitor cells, PC platelet concentrate, *Pb-MSCs* peripheral blood-derived mesenchymal stem cells, *oAECs* ovine amniotic epithelial cells, *COMP* cartilage oligomeric matrix protein, *COL3* collagen type III, *COL1* collagen type I, *TNMD* tenomodulin, *TNC* tenascin-C

### Umbilical cord blood-derived MSCs

Equine MSCs derived from the umbilical cord blood (UCB) or tissue (UCT) were first characterized by Koch et al. [34] and Hoynowski et al. [35]. Although autologous UCB-MSCs need a long initial culture to obtain a sufficient number of MSCs prior to use, allogeneic cultured cells can overcome this limitation as an alternative approach. Therefore, access to allogeneic UCB-MSC banking in analogy with a human can increase the chance of equine cell therapy [36]. In addition to the advantage of non-invasive collection, it has been demonstrated that expression of scleraxis (SCX) is similar to that of tendon*-*derived MSCs (TDSCs) in undifferentiated, monolayer-cultured at passage 3 [37].

### Tendon*-*derived MSCs

A current study has shown TDSCs (also known as tendon stem/progenitor cells) as an ideal cell type that displayed tendon-like phenotype and expressed the greatest level of tendon-related markers compared to other sources of MSCs for tendon regeneration [38]. Although using TDSCs showed promising outcomes [32], utilizing them might be limited due to donor site morbidity, inadequate cells that need a long period of culturing, and phenotypic drift during in vitro expansion. To overcome these limitations to some extent, different approaches such as using three-dimensional (3D) culture systems or the addition of growth factors have been reported [39, 40].

### Embryonic stem-like cells

Embryonic stem-like cells (ESCs) can provide a source of allogeneic cells for treating tendon injuries in horse. Unlike human and murine ESCs, equine ESCs have not been shown to form teratoma [41]. Although ESCs overcome some practical limitations of autologous MSCs, they require the destruction of an embryo for being isolated [42]. However, the clinical potential of ESC in the treatment of tendon injuries is revealed, and the absence of tumorigenic deviations of these cells remains to be studied in longer follow-ups [28, 43]. It has recently been suggested that the ethical and legal issues of ESCs for clinical application can be overcome by induced pluripotent stem cells (iPSCs). IPSCs maintain an epigenetic memory of their origin; it can adversely affect their differentiation potential. The generation of iPSCs from equine fibroblasts constitutes an important step toward the understanding of pluripotency in horse and a clinical tool in veterinary biomedicine [44, 45]. Moreover, their ability to differentiate into tendon cells has been demonstrated [5].

Taking together, equine MSCs derived from the adipose tissue, umbilical cord blood, and many other tissues are promising candidates in regenerative medicine. Implantation of MSCs can recruit the other MSCs or progenitor cells toward injury site since they produce a variety of cytokines and paracrine factors to improve the regeneration potential [46].

## Challenges of stem cell therapy in equine tendon injury

Although the application of stem cells for tendon healing is promising, some challenges should be considered in this field. Efficacy of equine MSC therapy is difficult to be evaluated, and it depends on the use of appropriate control groups, the severity and size of the lesion, time between injury and implantation, number of stem cell for implantation, models of tendinopathy (e.g., collagenase or surgical disruption), and opting for single or multiple injection. Intralesional injection of a cell suspension is the common approach since the tendon lesions are typically located in the center of SDFT [47]. Depending on the size and the severity of lesion, the number of stem cells and the volume of cell suspension should be estimated prior to injection. It has been suggested that numbers of MSCs from 10 to 50 × 10^6^ and the injection volume less than 1 ml are required to prevent damage to fibers due to compression [48]. It has been demonstrated that the best time for cell therapy is after the inflammatory phase [12]. The number of injection sites depends on the distribution of the injected cells, the type of the cells, and the level of damage observed ultrasonographically [16]. In addition, immunomodulatory effects of MSCs are dose and time dependent, so that different outcomes are reported after single or repeated injection [49–52]. There are some concerns about the use of direct injection of aspirated heterogeneous mixed cell, for instance a small number of stem cells in a large volume might disrupt remaining intact tendon tissue; therefore, concentration of aspirate would overcome this limitation to some extent [12]. Also, cell culture technique is helpful for re-implantation of large numbers of MSC [53]. Although transporting the cells to clinic, the handling step in clinic and injection process including sheer stress caused by needle wall, needle size, and the use of other tools for injection influence post-injection cell viability and differentiation potential by increasing the number of dead or damaged cells [54]. Twenty-four hours after injection, more of half of labeled cells are lost, which may enter the blood circulation due to damages in blood vessels by injection process and relocate to other injury sites [23, 55, 56]. Therefore, selection of the implantation technique as well as reliable techniques for tracking of transplanted cells is still challenging.

Though some studies have a control group, the limited sample size in horse studies and the inter-animal variability of the pathological conditions influence outcomes. Furthermore, some cases are mixed with other biological factors such as BM supernatant [17], autologous serum, platelet-rich plasma (PRP) [19], and genetically modified MSCs [29]. Extensive in vivo studies have been conducted on cell therapy for tendon injury in the horse (Table 1).

## Various strategies for in vitro tenogenic differentiation of equine stem cells

**Fig. 1:**
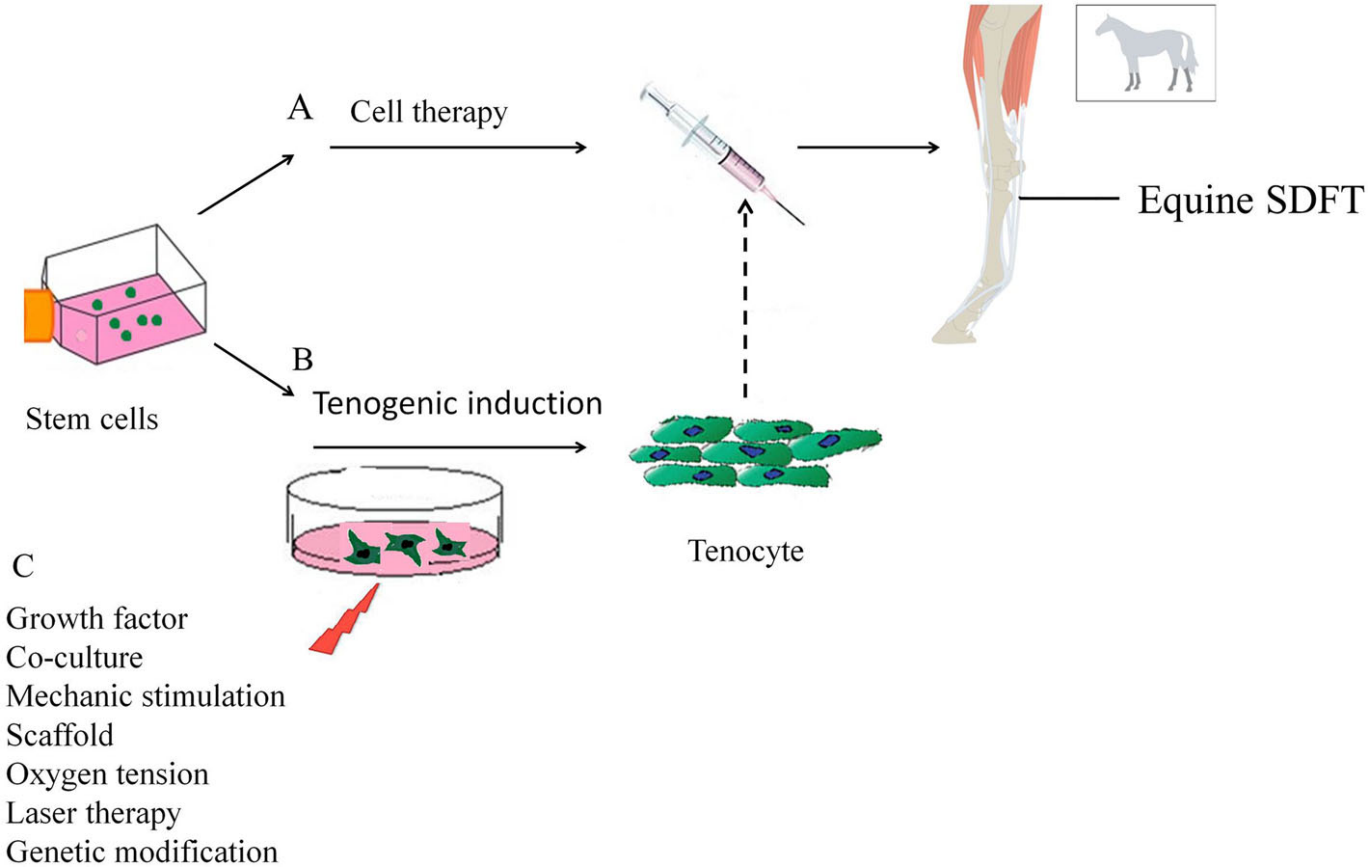
Schematic overview of the Cell therapy based on utilizing undifferentiated stem cells (**a**) or differentiated stem cells (**b**) through various strategies (**c**) for tendon injury in horse. Dash line indicated possible applications of differentiated stem cells under different strategies for tenogenic differentiation

The result of MSC therapy can be affected by the use of undifferentiated or differentiated MSCs for tendon repair since the risk of ectopic bone formation after undifferentiated MSC injection in tendon has been reported [57, 58]. To avoid the abovementioned risk, MSCs would be induced toward tenogenic differentiation before clinical application [30, 31]. Figure 1 demonstrates a schematic view on our understanding for improving the tendon regeneration potential in horse. In vitro differentiated stem cells could possibly result in faster regeneration after application [31]. In addition, it has been demonstrated that 24% of injected MSCs were retained at the site of injury after 24 h and most of the MSCs migrate from the site of injury after transplantation which might be directed to non-tenocyte differentiation in the in vivo condition [55]. Different strategies have been described to improve the ability of MSC and target sites to better respond to the homing stimuli and recruitment of stem cells respectively [59]. Here, we describe different strategies of recent findings that are often a combination of different strategies for tenogenic differentiation of equine stem cells.

### Growth factors

Growth factors (GFs) are important signaling molecules, involving tendon development and differentiation, which are produced at various stages of tendon healing [60]. The effect of exogenous addition of GFs to cell culture media, which trigger tenogenic differentiation, is influenced by the delivery of a single or multiple GF, incubation time, and cell type. Moreover, proper doses of GFs need to be determined in order to achieve better results. It should be noticed that small variations in concentrations of the GFs can result in considerably different effects [61]. In addition, they have a short half-life, which necessitates repeated dosing that poses costly challenge in their clinical application. Table 2 summarizes the results of in vitro experiments that investigated the role of GFs on tenogenic differentiation of various stem cells in horse.

**Table 2:** Summary of in vitro studies on tenogenic differentiation by growth factors in horse

Growth factor	Concentration	Other modification	Cell source	Outcome	Reference
TGFB3	20 ng/ml	2D and 3D cultures	Tenocytes and ESCs	Unlike tenocytes, ESCs upregulated tendon markers in 2D culture and showed synergic effect with TGFB3 and 3D; no cartilage or bone tissue deposition	[41, 62]
TGFB3	20 ng/ml	3D collagen gel	IPS	Reduced expression of tendon-related marker of iPSCs in 3D versus 2D culture	[5]
BMP12	50 ng/ml		AF-MSCs	Elongated and spindle-shaped; expressed *TNMD* and *DCN* genes	[63]
BMP12	50 ng/ml		BM-MSCs	Elongated tenocyte-like phenotype; expressed *TNMD* and *DCN* genes	[64]
BMP12	50 ng/ml		UCB-MSCs	Expression of *SCX*, *MKX*, *TNM*, *COL1*, and *DCN* by RT-PCR; expression of protein TNM, DCN	[65]
TGFB3, EGF2, bFGF2, IGF-1	10 ng/ml	LLLT	PB-MSCs	Supplementation with bFGF2 and TGFB3 upregulated expression of *EGR1*, and *DCN*; increased *TNC* with LLLT	[66]
PDGF-BB, IGF-1, bFGF, SDF-1 α, and GDF-5	5, 50, 5, 50 and 100 ng/ml respectively	Scaffold	Tenocytes	Effect of pairing IGF-1, GDF-5 rescue the tenocyte phenotype and gene expression profiles and driving proliferation	[67]
TGFB1, IGF-1, insulin	10 ng/ml and 50 ng/ml	2D and 3D cultures	Tenocyte	Pro-tenogenic effect with 3D culture system treated with GFs	[40]
GDF5, GDF6 and GDF7	10 ng/ml and 100 ng/ml	Strain stimulation; oxygen tension	AD-MSC	Expression of tendon-relevant genes were higher with an oxygen tension of 21%, tensile stimulation and supplementation with GDF5 or GDF 7	[68]
IGF-1	100 ng/ml	Acellular tendon matrix	BM-MSCs and TDSCs	COL and GAG syntheses were higher in TDSCs; no significant difference was observed in the expression of *COL1*, *COL3*, and *COMP* between BM-MSCs and TDSCs	[69]
TGFB3	2.5 ng/ml	Treated with Gremlin and SOST; nanofiber scaffold	ASCs	Increased tenogenic markers; decreased osteo-chondrogenic markers treated with T/G/S on nanofiber scaffold	Our unpublished data

*Abbreviations*: *TGF-β* transforming growth factor, *BMP* bone morphogenetic protein, *EGF* epidermal growth factor, *bFGF* fibroblast growth factor, *IGF-1* insulin-like growth factor, *PDGF-BB* platelet-derived growth factor-BB, *GDF* growth and differentiation factor, *SDF-1* stromal cell-derived factor-1, *EGR1* early growth response protein 1, *DCN* decorin, *COL3A1* collagen type III, *COL1A1* collagen type I, *TNMD* tenomodulin, *T/G/S* TGFB3/Gremlin/SOST, *LLLT* low-level laser therapy

### Co-culture system

Tendon regeneration can occur either via the ability of MSCs to differentiate into tenocytes within the tissue or via trophic factors produced by MSCs, or a combination of these two mechanisms [48]. The first study on co-culture in equine species was reported by Lovati et al. [70]. They demonstrated that indirect co-culture of equine BM-MSCs with tendon for 2 weeks could induce tenogenic differentiation. Probably, the paracrine factors released by tendon could be responsible for the observed effect.

Lange-Consiglio et al. [71] also highlighted the paracrine effect of possible mechanisms for tendon healing process. They investigated the effect of immunomodulatory of equine amniotic membrane-derived MSCs (AMCs) both in direct and indirect co-culture systems and demonstrated that AMCs inhibit the proliferation of equine peripheral blood mononuclear cells (PBMCs) after allogeneic stimulation in both culture systems. They suggested that secreted factors of the conditioned medium (CM) are responsible for the anti-proliferative effect; therefore, no cell-to-cell contact was required. Moreover, injection of AMC-CM in spontaneous tendon injuries in horse showed no adverse effect such as fibrotic, metaplastic, or mineralization. In addition, the re-injury rate was lower in comparison with untreated cases after 2 years.

A recent study demonstrated that co-culture of ovine amniotic epithelial cells (oAECs) with adult equine tendon started to aggregate and formed three-dimensional bundle structure after 28 days with fusiform-aligned cells, while oAECs cultured alone reached a confluent monolayer. Furthermore, analyses by reverse transcription polymerase chain reaction (RT-PCR) showed similar expression of *COL1*, *SCX*, and *COL3* in oAEC co-culture compared to ovine tendons [26].

### Various scaffold parameters

It is well-known that ECM not only provides a mechanical support for cells but also regulates cell behavior. In addition, cells are responsible for secreting ECM components; thus, ECM is dynamic [72]. Recently, in vitro studies, which have mimicked the dynamic nature of the ECM, have tried to improve scaffold designing to promote tenogenic differentiation of equine stem cells as described here.

#### Scaffold substrate materials and biomolecule presentation

A primary effort in the development of regenerative medicine is the choice of an appropriate biomaterial scaffold being able to mimic native ECM for providing an environment to speed healing or regeneration. Biologic scaffold materials generate chemotactic molecules through scaffold degradation process to the recruitment of stem cells. Not only that, they have shown to alter the local innate immune response, which contributes to tissue repair and scaffold remodeling [47]. Reed et al. [73] investigated the effect of three different substrates on tenogenic differentiation and showed equine UCB-MSCs and ASCs cultured on gelatin-coated plasticware, 30% matrigel or collagen-coated beads and treated with a different isomer of fibroblast growth factor (FGF) increased SCX expression on matrigel, especially in ASCs. However, the regulation of tenogenic gene expression in response to FGF stimulation is considerably different in the two cell types.

Interactions of growth factors and ECM in regulating the repair process are important. Many of these growth factors have been utilized either in the form of bound to the extracellular matrix (in the sequestered form) or freely soluble in it. Immobilizing the proteins is important to develop long-term tissue engineering solutions for controlling the growth factor delivery, particularly when increased diffusion or internalization of factors and reduced stability biomolecules are observed due to their short half-life [74, 75]. The effect of delivery method (e.g., soluble, sequestered) of five biomolecules on the behavior of equine tenocytes seeded on anisotropic collagen-glycosaminoglycan (CG) scaffold in tendon regeneration applications showed that sequestration can lead to a greater sustained bioactivity compared to soluble supplementation [40].

#### Three-dimensional scaffolds

As discussed previously, a critical drawback of prolonged in vitro culturing of tenocytes is the loss of differentiated function. It has been revealed that three-dimensional (3D) culture system prevents cellular de-differentiation to some extent. In this regard, Theiss and colleagues [40] showed that 3D microtissue system maintains the tenocyte phenotype in vitro. They also demonstrated that equine tenocytes retained a more differentiated state when scaffold-free micro tissue spheroids were embedded in collagen gels.

Barsby et al. [62] indicated that 3D culture enhanced tenogenic differentiation of equine ESCs seeded into 3D anchored collagen in comparison with 2D. In addition, equine tenocytes and ESCs are able to form constructs resembling artificial tendon by contraction of the matrix. Moreover, treatment with transforming growth factor (TGFB3) increased the initial rate of contraction and had a synergic effect on the upregulation of tendon-associated gene expression in 3D ESC culture, while the presence or absence of TGFB3 had no effect on contraction rate of tenocyte constructs. Although they did not compare tendon-related marker expression in 2D and 3D culture for equine tenocytes, it seems that 3D culture could keep the expression of tendon-associate proteins constant for long-term culture [41, 62]. Comparing the functional tendon differentiation of iPSCs with ESCs in 3D culture system by the same group showed that ESCs and iPSCs treated with TGFB3 in 2D culture system upregulated tendon-related genes; however, iPSCs delayed in comparison to ESCs. Furthermore, in contrast to ESCs, expression of tendon-associated genes with the exception of COL1 was not detected in iPSCs seeded on constructs and failed to generate artificial tendons. They suggested that one of the reasons is epigenetic differences between iPSCs and ESCs [5].

Decellularization of tendon tissue provides a 3D scaffold with a native ECM and a similar structure and topography to the tendon. Different protocols for decellularization of tendon tissue by using physical or chemical methods have been investigated [76]. Comparison of some tendon extracellular matrix markers of the cells isolated from equine bone marrow, tendon, and muscle on tendon matrix showed that *COL1* expression was similar among different cell sources and TDSCs expressed highest *COL3* expression [77].

#### Scaffold micro-nano structure

Mechanical properties of scaffold at the macro- and micro-scales are known to influence the cellular behaviors. Accordingly, 3D culture is important to prevent tenocytes de-differentiating within 2D culture. Recently, it has been revealed that phenotype of tenocytes is lost within 3D scaffold. Maintaining a high degree of anisotropy in scaffold to prevent altering cell fate due to loss of structural stability via cell-mediated contractile forces is a challenge in tendon tissue engineering [78]. The anisotropic CG scaffold with high crosslinking densities and small pore sizes indicated the increase in bioactivity of equine tenocytes and resistance to contraction as well as an increase and maintenance in expression of tenogenic markers for long-term culture [39].

The nanoscale topography is another factor in scaffold designing which indicated good results on tenogenic differentiation. Popielarczyk et al. [79] investigated the effect of topography on tenogenesis and showed that nanofiber topography alone can influence the tenogenic differentiation of equine BM-MSCs. Upregulation in the expression of tenogenic genes and production of ECM component was observed in aligned nanofiber scaffold with both a parallel and perpendicular oriented fibrous.

As mentioned previously, typical equine tendon lesions are located in the center of the SDFT surrounded by almost intact tendon tissue. Therefore, the choice of a scaffold, delivery of which into equine tendon injury is easy, should be considered; further studies are needed in the future.

### Mechanical stimulation

The mechanical stimulation is a major parameter in tendon biology. Conversion of mechanical stimulus into a biomechanical signal results in cell proliferation, differentiation, and ECM synthesis. Different physical environmental factors, from substrate stiffness to dynamic mechanical loading, in the form of static tension or cyclic axial stimulation, may regulate tenogenic stem cell differentiation [1, 80]. Depending on the stimulation regime, mechanical stress can induce tenogenic or osteogenic differentiation of stem cells. Raabe et al. [68] examined the influence of strain as the sole factor or in combination with other factors (GFs and O2 tension) in equine ASCs cultured on collagen I gel scaffold. The results of uniaxial tensile strain versus no mechanical stimulation showed tendon-like morphology with an alignment of cells and matrix in the collagen I gel construct. In addition, comparing the three cyclic strain (0, 3, and 5%) on tenogenic differentiation made by Youngstrom et al. [81], equine BM-MSCs seeded on decellularized tendon scaffold under 3% cyclic strain showed an increased expression of *SCX*, *COL1*, decorin (*DCN*), and biglycan, as well as increased ratio of relative *COL1* to *COL3*, and an increase in elastic modulus and ultimate tensile strength of construct. However, cyclic axial strain can also increase the expression of osteogenic markers. In this respect, equine ASCs were seeded on decellularized tendon matrix under static and 2% cyclic strain with different stimulation regime, which showed upregulation of expression of osteopontin, *COL3*, and *DCN* and downregulation of *COL1* in all of the groups compared to that of the monolayer control group. Although the expression of *SCX* at the last time point was upregulated slightly, a significant increase was observed under a short period of mechanical stimulation. They found that the difference between gene expression in their study and the findings of Youngstrom et al. is associated with the time points of gene expression analysis. They also showed that tendon matrix synthesis and tenogenic differentiation were under moderate mechanical stimulation regimes [82].

### Laser therapy

Low-level laser therapy (LLLT) is a modality to reduce inflammation and pain and to accelerate tissue healing. There is little literature on the controversial outcome of the use of LLLT for equine tendinopathy. The interpretation of in vivo outcomes is considerably difficult due to many intervening variables. Recently, several studies have investigated the in vitro effect of laser irradiation on the cellular behavior; it depends on laser light wavelength, energy density, and cell type [83, 84]. Irradiating equine PB-MSCs with a 660-nm wavelength laser indicated no significant difference in proliferation and differentiation versus the control group, although combination of some growth factors with LLLT arrested cell proliferation and enhanced tenogenic differentiation in comparison with the other group. The co-treatment of PB-MSCs with bFGF2 and TGFB3 without LLLT significantly increased the expression of early growth response protein-1 (EGR1) and *DCN*, while the synergistic effect of GFs with LLLT significantly increased expression of *EGR1*, *DCN*, and Tenascin C [66].

### Genetically modified cells

Several in vitro studies have investigated the role of gene products in tendon healing by gene delivery growth factors, transcription factors, and non-coding RNA into equine stem cells.

BMP12 has been established as a tenogenic growth factor, and a promising finding of equine stem cells treated with recombinant BMP12 protein is presented in Table 2. Furthermore, early cellular effects of equine tenocytes and BM-MSCs transfected with BMP12 and BMP2 were observed in response to BMP12. The upregulation of *COL1* and cartilage oligomeric matrix protein (COMP) expression was the greatest in tenocytes treated with BMP12 compared to BM-MSCs, and no mineralization detected in both cell types. It suggested that BMP12 gene delivery might induce early differentiation in early tendon healing [85].

The role of microRNA (miRNA) has been revealed in tendinopathy and tendon injury healing. In this regard, Millar et al. [86] indicated that the expression of *COL3* was upregulated in tendinopathy. By contrast, miR-29a expression was significantly downregulated. Additionally, in vitro transferring of miR-29a into equine tenocytes showed the reduced expression of *COL3*. Moreover, inhibition of miR-29a upregulated the expression of *COL3*. In fact, miR-29a plays an important role in the regulation of *COL3* expression in tendinopathy. Accordingly, the condition medium of AMCs decreases the pro-inflammatory genes and appears to demonstrate promising in vivo results for tendon healing. Indeed, miRNAs, identified in microvesicles secreted by equine AMCs, are responsible for these effects [87].

SCX is a well-known transcription factor in tendon development and differentiation, while it has been recently reported that SCX has a distinct role in different stages of development and in different cell types. Knockdown of *SCX* reduced the expression of *COL1*, *COMP*, and *SOX9* in fetal tenocytes, while it made no significant changes in the expression of their genes in adult tenocytes in 2D. Furthermore, adult tenocytes transfected with shSCX contracted the 3Dcollagen gel, while fetal tenocytes and ESCs failed to generate artificial tendon following *SCX* knockdown. SCX overexpression in fetal tenocytes, in which SCX had been knocked down formerly, reversed these effects [88].

As described above, the upregulation of expression of *SCX* was observed after physiological loading, but its mechanoresponse is not well defined. Recently, one study identified the novel role of SCX in modulating cytoskeletal tension. Equine tenocytes transfected with SCX siRNA decreased cytoskeletal stiffness by changing the focal adhesion-related gene expression and resulted in an inability to migrate on the soft surface [89].

### Oxygen tension

It is well known that oxygen (O_2_) tension depends on species, source of tissue, and other factors influencing the cell behavior. Although the physiological condition is hypoxic in some tissues, in vitro culture condition is routinely normoxic and needs further studies for standardization of the cell culture [90, 91]. The first analysis of the influence of oxygen tension on the behavior of equine MSCs showed that hypoxia reduces the proliferative capacity of cells, while it does not have any effect on the phenotype of cells, and it appears to keep them more undifferentiated [90].

Comparison of the influence of normoxic and hypoxic conditions (3% versus 21% O_2_ tension) on tenogenic differentiation of ASCs indicated that the cell morphology was more tendon-like under 21% O_2_, while the gene expression of the tendon-relevant markers revealed no significant differences. The gene expression of *COL1* was higher under 21% O_2_ than 3% O_2_. Cells were almost damaged under hypoxic conditions [68].

## Conclusion

Taken together, despite the wide range of studies, translating basic findings to clinical applications is limited; it is due to some concerns about the risk of bone, tumor, and scar formation. To fill the gap between experimental research and clinical applications, reliable and specific markers for the identification of tenocytes as well as conducting non-randomized studies with long-term follow-up periods are deemed necessary for further evaluation of the efficacy and safety of tendon injury. Finally, there are great resemblances between equine superficial digital flexor tendon and human Achilles tendon in the size of anatomical structure and load, function (energy store), pathophysiology of tendon injury, and the healing response under activity or traumatic rupture compared to other species [11, 92]. Moreover, considering the result of induced tendinopathy in equine species which reflects the conditions encountered in human, horse is accepted as an appropriate model in this area by research community and other authorities such as the US Food and Drug Administration (FDA) and the European Medicines Agency (EMA) [93]. Moreover, the high-level analogy between human and equine MSCs may have a great translational value for both species for future clinical aspects [93, 94]. As summarized in this review, utilizing tenogenically induced MSCs through pretreatment with bioactive compounds and applying other in vitro strategies may increase cell survival and the efficacy of cell therapy for tendon repair. Most of the success achieved in cell therapy in horses with core lesions in SDFT has been observed following the intralesional injection due to granulation tissue and the enclosed nature of core lesions that may have provided an appropriate scaffold. Therefore, for other forms of damaged tendon (eccentric lesions), future studies should optimize cell dose, time, and route of injection since accurate injection placement and retention of cells are more problematic [53]. In this case, the use of delivery vehicle such as different types of scaffold or self-organizing tendon (3D tendon-like tissue constructs) may improve stem cell retention at the site of injury with regard to ensuring that implantation of cells should occur within 24 h of resuspension in culture. In addition, it should be considered that the individual differences such as age, genetic factors, and donor health status affect the properties of MSCs [95]. Hence, the complete molecular analysis of MSCs in order to their modification seems to be highly necessary before the clinical application.

## Data Availability

The sources for the information discussed in this review can be obtained from the papers cited in the references.

## Abbreviations

AMCs: Amniotic membrane-derived MSCs
ASCs: Adipose tissue-derived MSCs
BM-MSCs: Bone marrow-derived mesenchymal stem cells
CM: Conditioned medium
ESCs: Embryonic stem-like cells
FGF: Fibroblast growth factor
GFs: Growth factors
iPSCs: Induced pluripotent stem cells
LLLT: Low-level laser therapy
oAECs: Ovine amniotic epithelial cells
TDSCs: Tendon*-*derived MSCs
TGFB3: Transforming growth factor
UCB: Umbilical cord blood
